# Differing Effects of Standard and Harsh Nucleic Acid Extraction Procedures on Diagnostic Helminth Real-Time PCRs Applied to Human Stool Samples

**DOI:** 10.3390/pathogens10020188

**Published:** 2021-02-09

**Authors:** Tanja Hoffmann, Andreas Hahn, Jaco J. Verweij, Gérard Leboulle, Olfert Landt, Christina Strube, Simone Kann, Denise Dekker, Jürgen May, Hagen Frickmann, Ulrike Loderstädt

**Affiliations:** 1 Department of Microbiology and Hospital Hygiene, Bundeswehr Hospital Hamburg, 20359 Hamburg, Germany; tanja1hoffmann@bundeswehr.org (T.H.); or hagen.frickmann@med.uni-rostock.de (H.F.); 2 Institute for Medical Microbiology, Virology and Hygiene, University Medicine Rostock, 18057 Rostock, Germany; hahn.andreas@me.com; 3 Laboratory for Medical Microbiology and Immunology, Elisabeth Tweesteden Hospital, 5042 AD Tilburg, The Netherlands; j.verweij@etz.nl; 4 TIB MOLBIOL, 12103 Berlin, Germany; gleboulle@tib-molbiol.de (G.L.); olandt@tib-molbiol.de (O.L.); 5 Institute for Parasitology, Centre for Infection Medicine, University of Veterinary Medicine Hannover, 30559 Hannover, Germany; christina.strube@tiho-hannover.de; 6 Medical Mission Institute, 97074 Würzburg, Germany; simone_kann@hotmail.com; 7 Infectious Disease Epidemiology Department, Bernhard Nocht Institute for Tropical Medicine Hamburg, 20359 Hamburg, Germany; dekker@bnitm.de (D.D.); may@bnitm.de (J.M.); 8 Department of Hospital Hygiene & Infectious Diseases, University Medicine Göttingen, 37075 Göttingen, Germany

**Keywords:** helminths, stool samples, consensus, nematodes, trematodes, cestodes, qPCR, test comparison

## Abstract

This study aimed to assess standard and harsher nucleic acid extraction schemes for diagnostic helminth real-time PCR approaches from stool samples. A standard procedure for nucleic acid extraction from stool and a procedure including bead-beating as well as proteinase K digestion were compared with group-, genus-, and species-specific real-time PCR assays targeting helminths and nonhelminth pathogens in human stool samples. From 25 different in-house and commercial helminth real-time PCR assays applied to 77 stool samples comprising 67 historic samples and 10 external quality assessment scheme samples positively tested for helminths, higher numbers of positive test results were observed after bead-beating-based nucleic acid extraction for 5/25 (20%) real-time PCR assays irrespective of specificity issues. Lower cycle threshold values were observed for one real-time PCR assay after the standard extraction scheme, and for four assays after the bead-beating-based scheme. Agreement between real-time PCR results after both nucleic acid extraction strategies according to Cohen’s kappa ranged from poor to almost perfect for the different assays. Varying agreement was observed in eight nonhelminth real-time PCR assays applied to 67 historic stool samples. The study indicates highly variable effects of harsh nucleic acid extraction approaches depending on the real-time PCR assay used.

## 1. Introduction

Although the global burden of helminth infections is still considerable [1,2,3,4,5,6,7,8,9,10,11], reliable microscopic diagnosis requires experience that is scarcely available in peripheral laboratories in settings of nonendemicity apart from reference centers. An investigator-independent, standardized screening option for both individual diagnosis and surveillance purposes is therefore desirable.

Multiple genus- and species-specific PCRs and real-time PCRs [12,13,14,15,16,17,18,19,20,21,22,23,24,25,26,27,28] have been introduced for helminths. A cox-gene-based group-specific real-time PCR approach targeting nematodes and cestodes has been described [29], as has a 28S-rRNA-gene-based approach targeting the trematode group [30].

While superior sensitivity of real-time PCR for protozoan parasites compared to microscopy is considered to be well established [31], reliability of diagnostic real-time PCR for helminths is believed to depend on the harshness of nucleic acid extraction from strong-shelled eggs or cuticle cells [32,33,34]. However, assessments of nucleic acid extraction schemes are usually performed only for specific helminth species, so it is difficult to draw general conclusions.

In the present study, a standard nucleic acid extraction scheme was compared with a harsh, bead-beating-based extraction protocol and applying multiple different helminth real-time PCRs. This was done in order to assess whether and to what extent increased sensitivity after harsh extractions is a general or a species-dependent effect.

## 2. Materials and Methods

### 2.1. Study Design and Sample Materials

#### 2.1.1. Study Design

The study was performed as a head-to-head comparison of two different nucleic acid extraction procedures regarding their effects on various diagnostic helminth real-time PCRs. Thereby, it was organized in a stepwise algorithm. In a first step, the test characteristics of the applied in-house helminth real-time PCRs were investigated. In a second step, the primary objective of the study, i.e., the comparative effects of nucleic acid extraction by either the QiaAMP DNA stool mini kit (Qiagen, Hilden, Germany) or a harsher, bead beating-based nucleic extraction procedure, was assessed. In a third step, the effects of the harsh bead beating-based nucleic extraction procedure on real-time PCRs targeting nonhelminth pathogens like bacteria and protozoa, which are also of diagnostic interest for the assessment of human stool samples in the medical diagnostic laboratory, were analyzed in comparison to the QiaAMP DNA stool mini kit-based nucleic acid extraction scheme. This was done to exclude harmful effects of such a harsh approach on the quality of DNA of less robust nonhelminth pathogens. The different steps are visualized in Diagram 1 and described in the following.

**Diagram 1:** Flowchart of the stepwise experimental approach.
1. Estimating the test characteristics of the applied in-house real-time PCRs:Specificity testing of the group-specific helminth real-time PCRs with helminth DNASensitivity and specificity testing of the group specific helminth real-time PCRs with DNA from stool samples positive in genus- and species-specific helminth real-time PCR after DNA extraction applying the QiaAMP DNA Stool Mini KitSensitivity and specificity testing of the in-house helminth real-time PCRs with DNA from stool samples positive for helminth eggs or larvae in microscopy after DNA extraction applying the QiaAMP DNA Stool Mini Kit2. Comparing the effects of nucleic acid extraction applying the QiaAMP DNA Stool Mini Kit and a harsher bead beating-based nucleic extraction scheme with:Historic residual stool samples which had been positive before in any helminth real-time PCR after nucleic acid extraction using the QiaAMP DNA Stool Mini KitStool samples from a Dutch external quality assessment scheme for helminths3. Comparative assessment of the effects of nucleic acid extraction applying the QiaAMP DNA Stool Mini Kit and a harsher bead beating-based nucleic extraction scheme on nonhelminth pathogens which might also be relevant in the medical routine diagnostic laboratory with:Historic residual stool samples which had been positive before in any bacteria real-time PCR after nucleic acid extraction using the QiaAMP DNA Stool Mini KitHistoric residual stool samples which had been positive before in any protozoa real-time PCR after nucleic acid extraction using the QiaAMP DNA Stool Mini Kit

#### 2.1.2. Specificity Testing of the Group-Specific Helminth Real-Time PCRs with Helminth DNA

As a first step, all group specific helminth real-time PCRs as described below were assessed with helminth DNA of *Trichinella spiralis*, *Fasciola hepatica*, *Toxocara canis*, *Toxocara cati*, *Angyostrongylus vasorum*, *Dipylidium caninum*, *Schistosoma mansoni*, and *Strongyloides ratti*, respectively, to exclude or confirm specificity limitations. Helminth tissue was provided from specimen collections from the institutions of the coauthors and the colleagues listed in the acknowledgements. Nucleic acid extraction was performed with the harsh bead beating-based procedure as described below.

#### 2.1.3. Sensitivity and Specificity Testing of the Group Specific Helminth Real-Time PCRs with DNA from Stool Samples Positive in Genus- and Species-Specific Helminth Real-Time PCR after DNA Extraction Applying the QiaAMP DNA Stool Mini Kit

As a second step, both sensitivity and specificity of the group specific helminth real-time PCRs were analyzed using 96 residual DNA volumes from stool samples of patients with real-time PCR-confirmed enteric helminth infections. As negative controls, 100 residual nucleic acid extractions from stool samples of German citizens without history of travel and thus a very low likelihood of being infected with helminths were applied. Nucleic acids had been extracted using the QiaAMP DNA stool Mini Kit (Qiagen, Hilden, Germany). The eluates had been stored at −80 °C prior to the assessment for time periods between few months and more than 10 years.

#### 2.1.4. Sensitivity and Specificity Testing of the in-House Helminth Real-Time PCRs with DNA from Stool Samples Positive for Helminth Eggs or Larvae in Microscopy after DNA Extraction Applying the QiaAMP DNA Stool Mini Kit

Prior to the nucleic acid extraction assessment comparisons, the applied in-house stool real-time PCRs for helminths as detailed below were tested with a small number of 37 nucleic acid extractions from microscopically positive stool samples from returning travelers with gastrointestinal symptoms after tropical journeys assessed at the Bernhard Nocht Institute for Tropical Medicine Hamburg, Germany. The samples had been collected within an eight-year interval (2006–2014) and extracted using the QiaAMP DNA stool mini kit (Qiagen). The eluates had then been stored at −80 °C. Native stool was no longer available from those samples, but the eluates were assessed in order to obtain an impression of baseline sensitivity of the applied real-time PCRs. Parasite microscopy that had been performed on the fresh stool samples was based on standard approaches including enrichment using sodium acetate–acetic acid–formalin solution (SAF) [35,36,37] and extended by Baermann’s method [38,39] in case of suspicion of *Strongyloides* infection. In fact, *Strongyloides* larvae had been seen after enrichment according to the Baermann method only. In detail, the 37 microscopically positive stool samples, i.e., samples in which at least one helminth egg or one helminth larva with potential pathogenic relevance for human patients had been microscopically observed, included had shown 29 nematode stages comprising 13 samples with *Strongyloides stercoralis* larvae; five with hookworm eggs; four with *Ascaris lumbricoides* eggs; three with *Trichuris trichiura* eggs; one with *A. lumbricoides* and *T. trichiura* eggs; as well as three with *Enterobius vermicularis* eggs; five samples with trematode eggs comprising one with small trematode eggs (suggestive of *Clonorchis sinensis*/*Opisthorchis viverrini*) and four with *Schistosoma mansoni* eggs; as well as three with *Hymenolepis nana* eggs as representatives of cestodes. Microscopical quantification had not been performed, i.e., the exact number of observed helminth eggs or helminth larvae per assessed microscopical slide had not been recorded. In the investigators’ experience, however, helminth loads in travel returnees are usually low.

#### 2.1.5. Comparing the Effects of Nucleic Acid Extraction Applying the QiaAMP DNA Stool Mini Kit and a Harsher Bead Beating-Based Nucleic Extraction Scheme

After this, the study was conducted as a head-to-head comparison of nucleic acid extractions using 67 residual materials from stool samples that had shown positive real-time PCR signals for helminths in previous diagnostic assessments at the Bundeswehr Hospital Hamburg, Department for Microbiology and Hygiene, external site at the Bernhard Nocht Institute for Tropical Medicine Hamburg, Germany. Those stool samples had originally been obtained for diagnostic screening purposes from soldiers and policemen returning from tropical deployments, migrants travelling under poor hygiene conditions, and study participants from resource-poor tropical settings over the course of 13 years (2006–2019). No microscopic results were available for those assessed stool samples. Both bead beating-free and bead beating-based nucleic acid extractions as detailed below and residual stool samples had been stored at −80 °C prior to further assessment. Ethical clearance allowed only the anonymous use of residual sample materials for test comparison purposes in a fully anonymized way. Accordingly, no detailed patient-related information can be presented. In addition to the 67 diagnostic samples used, 10 stool samples from a Dutch external quality assessment scheme for helminths as described elsewhere [40] were included in the comparison. From the residual sample collection in Hamburg, a further 67 residual materials from stool samples that had shown positive real-time PCR signals in nonhelminth real-time PCRs were chosen to assess the effects of both nucleic acid extraction schemes on real-time PCRs targeting bacterial and protozoan pathogens.

#### 2.1.6. Effects of Bead-Beating-Based Nucleic Acid Extraction on Nonhelminth Real-Time PCR Targets

To assess the effects of harsh bead-beating-based nucleic acid extraction on nonhelminth real-time PCR targets, the 67 residual stool samples with positive in-house real-time multiplex real-time PCR results for the enteroinvasive bacteria *Salmonella* spp., *Shigella* spp./enteroinvasive *Escherichia coli* (EIEC), *Campylobacter jejuni*, or *Yersinia* spp. [41] as well as the enteropathogenic protozoa *Entamoeba histolytica*, *Giardia duodenalis*, *Cryptosporidium* spp., or *Cyclospora* spp. [28] were subjected to bead-beating-based nucleic acid extraction with subsequent repetition of the real-time PCRs. Similar as described above for the helminth assessments, the applied residual DNA samples had been stored frozen at −80 °C over the course of 13 years (2006–2019) as well.

### 2.2. Nucleic Acid Extraction

For the initial diagnostic assessment, nucleic acid from all samples had been extracted using the QiaAMP DNA Stool Mini Kit (Qiagen, Hilden, Germany) as described by the manufacturer without particular focus on specialized procedures for parasite DNA (later referred to as “without bead beating”). If any helminth real-time PCR signal had been detectable in the initial diagnostic process and residual stool material was still available stored at −80 °C, nucleic acid from these residual materials were extracted using polyvinylpyrrolidone pretreatment, bead-beating using garnet beads (0.7–1.2 mm, Biolabproducts, Bebensee, Germany) with a TissueLyser LT device (Qiagen) for 3 min at 30 s^−1^, proteinase K digestion, and QiaAMP spin column extraction (Qiagen) as described previously [33]. This procedure is later referred to as “with bead beating”. Identical elution volumes as in the QiaAMP DNA Stool Mini Kit-based approach were achieved. To ensure sufficient sample volumes for the real-time PCR assays, photometric assessment of DNA concentrations within the samples using a Pico 100 Picodrop Microliter Spectrophotometer (Picodrop Ltd., Hinxton, UK) according to the manufacturer’s instructions was only performed from remaining residual DNA samples after the real-time PCRs had been completed.

### 2.3. Helminth Real-Time PCR Protocols

The total of 25 genus- and species-specific helminth real-time PCRs used comprised in-house real-time PCRs targeting *Ancylostoma* spp., *A. lumbricoides*, *E. vermicularis*, *H. nana*, *Necator americanus*, *Schistosoma* spp., *S. stercoralis*, *Taenia saginata*, *T. solium*, and *T. trichiura* as well as commercial real-time PCRs (Tib MolBiol, Berlin, Germany, product codes of the kits: MDx_64-0710-96, MDx_50-0705-96, MDx_50-0714-96, MDx_58-0707-96, MDx_64-0709-96, MDx_61-0708-96, MDx_58-0639-96) targeting *Ancylostoma* spp., *A. lumbricoides*, *E. vermicularis*, *H. nana*, *N. americanus*, *Schistosoma* spp., and *S. stercoralis*, as recently described [28]. Additionally, specific multi-copy-target real-time PCRs for *S. mansoni* complex [42] and *S. haematobium* complex [43], which were originally designed for application with blood samples, were used as detailed previously [42,43] with DNA from stool. Group-specific helminth real-time PCRs targeting nematodes, trematodes, and cestodes were applied as described [29,30] as simplex assays. In addition, group-specific assays for nematodes, trematodes, and cestodes targeting the 18S-rRNA-gene were run (details on the group-specific assays are provided in the Table 1 and Table 2). Thereby, genus- and species-specific real-time PCRs were run on RotorGene Q cyclers (Qiagen, Hilden, Germany), group-specific real-time PCRs on RotorGene 6000 cyclers (Qiagen). Real-time PCRs from eluates after different nucleic acid extraction procedures were not continuously assessed in parallel in the same runs but were distributed on the runs without a specific order.

### 2.4. Inclusion and Exclusion Criteria

Samples were included if at least one positive pathogen-specific real-time PCR result had been observed during routine diagnostic assessment. There were no exclusion criteria, so samples were also included if residual sample material was insufficient for all assessments.

### 2.5. Statistical Assessment

Due to the low sample count of this proof-of-principle assessment, statistical approaches were basically descriptive. In detail, Cohen’s kappa was calculated to assess the agreement between the extraction schemes for each individual real-time PCR target. The interpretation standards as described [44] with the categories poor (below 0.00), slight (0.00–0.20), fair (0.21–0.40), moderate (0.41–0.60), substantial (0.61–0.80), and almost perfect (0.81–1.00) were applied. Significance for differences regarding the qualitative results, i.e., positive or negative real-time PCR results, was calculated by applying tests for proportions. Normal distribution of the Ct (cycle threshold) values was assumed. This assumption was proven applying Shapiro–Wilk testing with Bonferroni correction for multiple testing. There was no evidence for rejecting the assumption of a normal distribution of the Ct values. For the descriptive comparison of Ct values, the unpaired T-test was applied without correction for multiple testing. Welch’s adjustment for unequal variances between the groups was applied if the hypothesis of equal variances was rejected (significance level 0.05). Unpaired T-testing was chosen as a consequence of different processing of the assessed patient materials. In detail, pretreatment of the patient materials, i.e., the nucleic acid extraction procedure, is a prerequisite to obtain analyzable samples. In turn, the sample itself is not assessed first with one method and then again with the other method, but rather two samples are generated from one patient material by different pretreatment approaches. Only then, they represent analyzable samples by themselves. Accordingly, they were considered as two different samples, favoring the use of unpaired testing. The calculations were performed using the software Stata/IC 15.1 for Mac 64-bit Intel (College Station, TX, USA).

### 2.6. Ethical Clearance

Ethical clearance, provided for blinded use of residual materials for test comparison and evaluation purposes, was granted on 11 March 2019 by the ethics committee of the Medical Association of Hamburg, Germany (registration number WF-011/19) in line with national laws without requirement for informed consent. The authors assert that all procedures contributing to this work comply with the Helsinki Declaration of 1975, as revised in 2008.

## 3. Results

### 3.1. Results of the Group Specific Helminth Real-Time PCRs with Helminth DNA

When applied with helminth DNA, individual cross-reactions of the group specific helminth real-time PCRs were seen. Such cross-reactions were associated with comparably high cycle threshold (Ct) values. Details of the Ct values of observed reactions and cross-reactions with nucleic acid extractions of helminths are shown in Table 3.

### 3.2. Sensitivity and Specificity Testing of the Group-Specific Helminth Real-Time PCRs with DNA Residuals from Stool Samples without Available Microscopic Results

Applied with 96 residual DNA samples extracted from stool of patients with previous real-time PCR-based diagnosis of enteric helminth infections, imperfect sensitivity and specificity of the group specific helminth real-time PCRs was calculated. In detail, application of the group-specific real-time PCRs with samples positive in genus- or species specific helminth real-time PCRs (in-house protocols according to [28]) after QiaAMP DNA Stool Mini Kit-based extraction without microscopic control gave the results as detailed in the Table A1 of Appendix A and summarized in Table 4. Of note, the group specific real-time PCRs for nematodes, trematodes, and cestodes did not show any cross-reactions with stool samples of 100 German individuals without any history of travel, making intestinal carriage of helminths highly unlikely, suggesting acceptable overall specificity of those assays.

### 3.3. Sensitivity Assessment with in-House Real-Time PCRs for Stool and Standard Nucleic Acid Extractions from Microscopically Positive Stool Samples

As assessed using the nucleic acid residuals of the microscopically positive samples (*n* = 37), specificity of the in-house stool real-time PCRs for helminths after QiaAMP DNA Stool Mini Kit-extraction was generally good (96.7–100%). In contrast, there was a broad range of sensitivity ranging from 20.7% to 100% over the different in-house real-time PCRs for stool. Details of the assessments with the microscopically positive samples including cycle threshold values are provided in Table 5. Insufficiency of sample volumes did not allow the inclusion of the *Schistosoma*-specific blood real-time PCRs and the commercial assays. So, this quality control assessment had to be restricted to the real-time PCR assays as shown in Table 3 and as reported previously [28]. Afterwards, the sample volumes were gone.

### 3.4. Comparison of Qualitative and Quantitative Results of Helminth Real-Time PCRs after the Two Compared Nucleic Acid Extraction Procedures

More positive results following bead-beating-based nucleic acid extraction as suggested by significance levels *p* < 0.05 were observed for in-house and commercial real-time PCR targeting *A. lumbricoides* and *N. americanus* as well as for commercial real-time PCR targeting *H. nana*. If Bonferroni’s correction is applied [45], however, the significance is challenged. A wide range of agreement in line with the definitions applied was observed between the compared nucleic acid extraction schemes, ranging from almost perfect agreement for the cox-gene-based pan-cestode real-time PCR, both the 18S and the 28S rRNA-gene based pan-trematode real-time PCRs, both the in-house and the commercial *Ancylostoma* spp. real-time PCRs and *Schistosoma* spp. real-time PCRs, and the in-house *H. nana* real-time PCR; through substantial agreement for the 18S rRNA gene-based pan-cestode real-time PCR, both the 18S rRNA gene- and the cox-gene-based pan-trematode real-time PCRs, the in-house *T. solium* and *S. mansoni* real-time PCRs, and the commercial *S. stercoralis* real-time PCR; moderate agreement for the in-house *T. trichiura* real-time PCR and the commercial PCRs targeting *A. lumbricoides*, *N. americanus*, and *H. nana*; fair agreement for the in-house *N. americanus* real-time PCR, and both the in-house and the commercial real-time PCRs targeting *E. vermicularis*; slight agreement for the in-house *S. stercoralis* real-time PCR; and poor agreement for the in-house *A. lumbricoides* real-time PCR. Focusing on the quantitative assessment based on Ct-values, there was a variable pattern with significantly lower Ct-values after the nucleic acid extraction without bead-beating for the in-house *T. solium* real-time PCR, as well as after the bead beating-based nucleic acid extraction for the commercial real-time PCRs targeting *A. lumbricoides*, *N. americanus*, *H. nana*, and *Schistosoma* spp. Again, the significance for Ct-value differences of the in-house *T. solium* real-time PCR and the commercial *A. lumbricoides* would be challenged by application of Bonferroni’s correction [45]. For all other real-time PCRs, either there was no significant difference or the calculation was impossible because of a lack of positive test results. Relevant sample inhibition was not observed with either extraction scheme. Details are shown in Table 6, Table 7 and Table 8.

The low number of external quality assessment scheme samples [40] did not allow an independent statistical assessment for those samples. With those samples, however, positive real-time PCR signals in case of discordant results were observed after bead-beating-based extraction only. A small proportion of Ct-values showed identical results irrespective of the extraction scheme within a ±1 Ct step range. A larger proportion showed lower Ct values after bead-beating-based extraction compared with the QiaAMP DNA Stool Mini Kit-based extraction. Convincing significance, however, could not be shown for differences in the Ct-value-distribution between the quality assessment scheme samples and the other samples. In detail, the distribution of the Ct values for all real-time PCRs with at least three recorded Ct values per sample group was evaluated. The Kolmogorov–Smirnov test was used, which tests the empirical distribution in the sample groups for difference. The null hypothesis was that the distribution of the Ct values of both samples was identical. The required level of significance was 0.05. For seven real-time PCR assays for which the abovementioned conditions were fulfilled, *p* values were 0.046, 0.518, 0.514, 0.080, 0.426, 0.431, and 0.334, respectively.

### 3.5. Comparison of Qualitative and Quantitative Results of Nonhelminth Real-Time PCRs after the Two Compared Nucleic Acid Extraction Procedures

Focusing on the qualitative results of the eight in-house real-time PCRs targeting bacteria and protozoa in 67 samples, significantly more positive results were found for *Shigella* spp./EIEC after bead beating-based nucleic acid extraction. No significant difference was detectable for any other parameter. Again, there was a wide spectrum of agreement, with substantial agreement for the in-house real-time PCRs targeting *Salmonella* spp., *C. jejuni*, and *G. duodenalis* and slight agreement for *Shigella* spp./EIEC and *Cyclospora* spp. For the other parameters, numbers of positive results were insufficient to allow the calculation of Cohen’s kappa. Significant difference in recorded Ct-values were never observed for the nonhelminth parameters. Details are shown in Table 9.

### 3.6. Photometric Assessment of DNA Concentrations within the Samples

After performing all real-time PCR assessments, sufficient residual sample volumes for photometric DNA quantification were available for 39 nucleic acid extractions without bead beating and for 77 nucleic acid extractions with bead beating as well as for 96 historic samples and 20 fresh external quality assessment scheme samples, respectively. There was no statistically significant difference between the DNA-concentrations in comparison of fresh external quality assessment scheme samples and historic samples (mean of 20.0 ng/µL for fresh quality control assessment scheme samples, mean of 23.0 ng/µL for historic samples, *p* = 0.99), while bead beating-based nucleic acid extraction (mean of 16.2 ng/µL) yielded about half as much DNA as nuclei acid extraction without bead beating (mean of 35.0 ng/µL) (*p* = 0.0003). *p* values were calculated using the Mann–Whitney U-test.

## 4. Discussion

The study was performed to compare performance characteristics of two different nucleic extraction procedures for diagnostic real-time PCR approaches targeting helminths. A number of genus- and species-specific in-house helminth real-time PCR approaches [28,42,43] supplemented by some commercial assays [28] were applied. Among the in-house assays were both previously described [29,30] and newly introduced group-specific helminth real-time PCRs targeting nematodes, trematodes, and cestodes. With both microscopically positive and negative samples (details are shown in Table 4 and Table 5 and Appendix A), these group-specific real-time PCRs showed imperfect sensitivity and specificity, predominantly with sensitivity issues. This makes them particularly interesting for potential improvement following the comparison of nucleic acid extraction procedures. Due to the low number of available microscopically positive samples and due to the detection limits of microscopy, recorded sensitivity and specificity values have to be interpreted with care. Accordingly, the study was focused on the comparison of sensitivity as affected by the mode of nucleic acid extraction only.

As well as a standard approach for nucleic acid extraction from stool samples, a more sophisticated approach including bead-beating and proteinase K-digestion was applied as suggested in a recent publication [33]. Most interestingly, however, in the present study this harsher extraction scheme was not generally superior. Instead, there were different reaction patterns depending on the real-time PCR assay used. In detail, benefits were seen for robust eggs of *A. lumbricoides* but also for fragile *N. americanus* eggs. For *T. trichiura*—a nematode with very robust eggs [33]—effects were less obvious, which is in contrast with previous works [33,34]. Nonsignificant tendencies of stronger influence of the bead-beating-based extraction scheme in standardized external quality assessment scheme samples—for which an independent statistical evaluation had low power due to the low number of available specimens—suggest likely but nonsignificant effects of sample age of the assessed sample collection. However, even within the external quality assessment scheme specimens, several samples showed identical Ct-values irrespective of the chosen nucleic acid extraction procedures. In contrast, considerable shifts of several decadic logarithmic steps were observed for other samples. The reasons for this observed inconsistency are unclear.

Of note, however, considerable differences between real-time PCR results following the different extraction schemes suggest detections at the threshold of diagnostic sensitivity. Close to the detection threshold, positive results necessarily tend to become stochastic phenomena. There was a marked dominance of lower Ct-values after the bead-beating-based extraction scheme for the commercial test assays compared to the in-house assays. The reasons for this remain unclear: different sensitivity of the assays might theoretically play a role here. However, a previous assessment [28] did not suggest marked differences between the in-house and the commercial real-time PCR approaches. At least for the Ct-values with the commercial real-time PCR assays, superiority of the bead beating-based extraction can be considered likely. In contrast, the results are less conclusive for the in-house approaches. For example, when looking specifically at the samples from the quality control assessment scheme (data not shown), the in-house assays also showed more positives with considerably lower Ct-values. An exception was *T. trichiura*, for which the recorded Ct-values were similar. However, those differences are mostly lost when assessing the whole sample collection.

Interestingly, however, not even the commercial assay showed unambiguous difference for the robust eggs of *T. trichiura*, which is in contrast to previous results [33,34]. As differences in results depending on the producer of the applied beads are known from previous assessments [33], minor differences in the production and resulting microstructure of the garnet beads might be a potential reason for this striking difference. This is an issue that might make the standardization of bead-beating extraction for helminth real-time PCRs challenging if suppliers are not interchangeable. The results with the external quality assessment scheme samples (data not shown) at least suggest a tendency toward more positive results and lower Ct-values after bead-beating-based extraction. So, the particular situation of working with historical samples may provide an explanation for this observed contradiction with previous studies as well, although the precise history of the external quality assessment scheme samples was unknown as well.

Considerable, target-dependent, variability in agreement of the results following the different nucleic acid extraction strategies was observed for the real-time PCRs with nonhelminth targets. Significantly more detection of *Shigella* spp./EIEC after the bead-based nucleic acid extraction strategy was counteracted by only slight agreement, so this result is difficult in interpret. Significant differences in the measured Ct-values were never observed, however.

The present study has a number of limitations. First, the samples that were used for the extraction comparison were not assessed microscopically. So, definite microscopic proof of the presence of pathogens was not feasible. Secondly, due to the rarity of occurrence of helminth-positive sample materials, the overall numbers of samples analyzed was low. Thirdly, and for the same reasons, the ages of the samples used varied between a few months and about 10 years, although frozen storage at −80 °C makes quantitatively relevant nucleic acid degradation less likely. Fourthly, there was no homogenization step of the initially provided stool samples, making uneven distributions of parasites in stool likely. Fifthly, real-time PCRs after different extractions were not consistently performed in parallel in the same run, and low volumes of residual sample materials did not allow repeated testing. So, the comparison indeed reflects routine-like conditions. Sixthly, due to limited available sample volumes, photometric assessments of the DNA concentration within the eluates could be performed only for a subset of the samples included in the study. Accordingly, effects to varying DNA concentrations after the different extraction schemes could not be analyzed for all samples. However, an exploratory investigation with the available residual sample materials did not indicate significant influence of sample age on measured DNA concentrations. Although the extraction scheme without bead beating yielded about twice as much DNA compared to the extraction scheme with bead beating, an average factor of two will only have minor influence on measured Ct values in real-time PCR. Seventhly, individual fecal samples contain variable concentrations of inhibitory components [46], potentially resulting in heterogeneity to the set of samples and the outcome of real-time PCR testing in case of uneven distribution within the sample due to the missing homogenization step. However, inhibition control real-time PCR based on a Phocid Herpes DNA sequence as described elsewhere [47] did not suggest relevant inhibition issues (data not shown). Eighthly, only frozen samples with potential effects of freezing on DNA release from helminth cells could be assessed. Ninthly, only human stool samples were included in the assessment. The analysis of other sample materials would have been interesting as well but was beyond the scope of this assessment.

In spite of these limitations, the assessment indicated that the harsh nucleic acid extraction scheme could indeed provide considerable shifts in Ct-values for several samples and also provided tendencies of higher sensitivity. However, the observation of Ct-value shifts is inconsistent. Especially with historic sample materials, which may play a role in retrospective epidemiological assessments rather than for routine diagnostic applications, the beneficial effects of the harsh extraction approach are less obvious than in previous reports assessing fresh samples. Nevertheless, early international external quality assessment trials [40] have suggested the applicability of real-time PCR for the diagnosis of helminths. Accordingly, broader application of such strategies is to be expected in the future, making studies on further optimization of pre-analytic conditions desirable.

## 5. Conclusions

This nucleic acid extraction comparison indicated inconsistent effects of the analyzed harsh nucleic acid extraction scheme on helminth real-time PCRs. There were only tendencies for beneficial effects of bead-beating-based extraction as observed with the samples from the external quality assessment scheme. If complex nucleic acid extraction schemes are infeasible for logistical reasons in diagnostic or study settings, sensitivity constraints may result. With historical samples in retrospective assessments, any beneficial effects of harsh nucleic acid extraction may be less pronounced than observed with fresh sample materials in the diagnostic routine in previous studies.

## Figures and Tables

**Table 1:** First real-time PCR platform with consensus real-time PCRs for nematodes, trematodes, and cestodes.

Oligonucleotide	Oligonucleotide Name	SEQUENCE	Reference
Nematode consensus real-time PCR 1			
Forward Primer	pan_nematode_cox1_692F	5′-TGT-CTT-TAC-CWG-TTT-TRG-CTG-G-3′	[29]
Reverse Primer	pan_nematode_cox1_835R	5′-CCG-AAA-GCA-GGY-AAA-ATH-ARA-A-3′	
Probe	pan_nematode_cox1_795P	5′-FAM-TCA-RCA-TTT-RTT-TTG-RTT-TTT-TGG-TCA-TCC-BHQ1-3′	
Trematode consensus real-time PCR 1			
Forward Primer	pan_trematode_28S_2F	5′-AGG-CAA-TGT-GGT-GTT-YAG-GT-3′	[30]
Reverse Primer	pan_trematode_28S_168R	5′-CAC-AAA-CAA-CCC-GAC-TCC-AA-3′	
Probe	pan_trematode_28S_T	5′-FAM-TGG-CCC-AND-GAG-GGT-GAA-AGG-C-BHQ1-3′	
Cestode consensus real-time PCR 1			
Forward Primer	pan_cestode_cox1_82F	5′-TGG-GTT-ATT-GTT-TGC-TAT-GTT-TTC-WA-3′	[29]
Reverse Primer	pan_cestode_cox1_209R	5′-CCC-CTA-TTA-TCA-TAG-TAA-CMG-AAC-TAA-A-3′	
Probe	pan_cestode_cox1_143P	5′-FAM-ATG-TTT-ACG-GTT-GGG-TTR-GAT-GTK-AAG-BHQ1-3′	
Combined positive-control plasmid sequence insert	Eco-R1-restriction-site-TTATTGGTTTTGTCTTTACCTGTTTTAGCTGGTGCTATTACTATGTTGTTAATTGATCGTAATTTTAATGGTTCTTTTTTTGATCCTAGTTTTGGTGGTAATCCTTTGATTTATCAGCATTTGTTTTGGTTTTTTGGTCATCCAGAAGTTTATATTTTAATTTTACCTGCTTTCGGTATTATTAGT-Eco-R1-restriction side-TTGGCTTTTATGGGTTATTGTTTGCTATGTTTTCAATAGTATGTTTAGGAAGAAGTGTGTGAGGACATCATATGTTTACGGTTGGGTTAGATGTTAAGACGGCTGTATTTTTTAGTTCTGTTACTATGATAATTGGAGTGCCTACGGG-Eco-R1-restriction-site-ATTGGTCACTAGGCAATGTGGTGTTCAGGTCGTTCCGCGGAGGTGCTGCTCCATTCCAAGTCCAGCAATGAGTACGGTAATGCTGACATGGCCCAAAGAGGGTGAAAGGCCCGTTGGGGTGGAGAGGCAGAAATGACAGCACCTTCCTGGATAGACCTTGGAGTCGGGTTGTTTGTGAATGCAGCCCAA-Eco-R1-restriction-site.		

The real-time PCRs were run in 20 μL reaction volumes containing 10 μL HotStar master mix (Qiagen, Hilden, Germany), 5.0 mM total MgCl_2_, 5 × 10^−7^ mol of each primer, 2 × 10^−7^ mol probe, and 4.0 μL DNA eluate on a RotorGene 6000 cycler (Qiagen). The steps were activation at 95 °C for 15 min (minutes) followed by 55 cycles of 45 s (seconds) denaturation at 95 °C and 45 s annealing and elongation at 55 °C. After this, the tubes were cooled to 40 °C for an additional 20 s before the run was finished.

**Table 2:** Second real-time PCR platform with consensus real-time PCRs for nematodes, trematodes, and cestodes.

Oligonucleotide	Oligonucleotide Name	Sequence	In-Silico Coverage	Genbank Accession Numbers
Nematode consensus real-time PCR 2				
Forward Primer	Nem1Gr1-6--7-Go S	5′-GAA-TYC-CTA-GTA-ART-GTG-AGT-CAT-C-3′	As detailed for the sum of the reverse primers	n.a.
Reverse Primer	Nem1Gr2 A	5′-GCC-TCT-SGA-TAT-TGC-TCA-GT-3′	*Strongyloides stercoralis*, *Strongyloides fuelleborni*, *Parastrongyloides trichosuri*	AB923885.1, LM523351.1, AB453320.1, AB453322.1, AB821045.1
	Nem_Ancylo B	5′-CTC-GAT-ATA-GCA-GGC-CGA-3′	*Ancylostoma caninum*, *Ancylostoma duodenale*, *Ancylostoma ceylanicum*	AJ920347.2, MH508247.1, EU344798.1, DQ464371, MH508245.1, LC036567.1
	Nem1X B	5′-GCC-TCG-AAA-CAG-CAG-TCY-SC-3′	*Enterobius vermicularis*, *Dracunculus medinensis*, *Dracunculus lutrae*, *Dracunculus insignis*, *Brugia malayi*, *Dirofilaria repens*, *Loa*, *Anisakis simplex*, *Ascaris lumbricoides*, *Baylisascaris schroederi*, *Baylisascaris ailuri*, *Baylisascaris transfuga*, *Baylisascaris procyonis*, *Contracaecum multipapillatum*, *Toxacara cati*, *Toxocara canis*, *Ancylostoma caninum*, *Ancylostoma duodenale*, *Ancylostoma ceylanicum*, *Angiostrongylus cantonensis*, *Angiostrongylus chabaudi*, *Angiostrongylus costaricensis*, *Angiostrongylus vasorum*, *Necator americanus*, *Oesophagostomum aculeatum*, *Trichostrongylus colubriformis*, *Gongylonema pulchrum*	AB626660, HQ646164, JF934731.1, KF770013.1, KF770015.1, JF934737.1, AY947719.1, AF100621, XM_001893642.1, AF036588.1, AB973229, MG657262.1, MH981971.1, AB973229, XR_002251421, MF072711.1, LN600407.1, U94366.1, JN256992.1, JN256991.1, U94369.1, KU050692.1, MG696302.1, EF180059.1, JN256973.1, AF036608.1, U94382.1, AJ920347.2, MH508247.1, EU344798.1, DQ464371, MH508245.1, LC036567.1, AY295804.1, KX378963.1, KX378964.1, AJ920365.1, AJ920348.1, AY295811.1, AB677956.1, AJ920350.1, AB646055.1, LC388753.1, AB495401.2
	Nem1Gr9 B	5′-CGG-CAT-CGG-TCC-AAA-3′	*Trichuris trichiura*	GQ352553.1, GQ352553.1, GQ352554.1
	Nem1Gr10 B	5′-CTA-CTG-GCG-CYA-GTC-AAA-A-3′	*Trichinella britovi*, *Trichinella nativa*, *Trichinella nelsoni*, *Trichinella pupae*, *Trichinella spiralis*	AY851257.1, KP307966, AY851261.1, AY851263.1, KU725991.1
Probe	Nem1 TM	5′-FAM-TAC-KTC-CCT-GCC-MTT-TGT-ACA-CAC-C-BHQ1-3′	As detailed for the sum of the reverse primers	n.a.
Trematode consensus real-time PCR 2				
Forward Primer	Trem1 F	5′-WGA-GGC-TCC-GTA-ATT-CGA-3′	As detailed for the sum of the reverse primers	n.a.
Reverse Primer	Trem1 Gr1 R	5′-TGC-GAY-CGC-ACK-ACC-C-3′	*Schistosoma intercalatum*, *Schistosoma japonicum*, *Schistosoma mansoni*, *Schistosoma mekongi*	DQ354363.1, U42564.1, AY157226.1, JF721335.1, U65657.1, X53047.1, XR_001974584.1, AY157228.1
	Trem1Gr3-6 R	5′-CRY-AGC-CAT-SCG-ACC-C-3′	*Fasciola gigantica*, *Gastrodiscoides hominis*, *Paragonimus westermani*, *Nanophyetus salmincola*, *Paragonimus vietnamesi*, *Paragonimus kellicotti*	AJ004804.1, MF077354.1, AJ011942.1, JX678223.1, KF781291.1, AY628372.1, AJ287556.1, AY222140.1, AY222138.1, KX990282.1, LT855189.1, HQ900670.1
Probe	Trem1 TM	5′-FAM-YCA-ACT-ACG-AGC-TTT-TKA-ACT-GCA-RCA-ACT-BHQ1-3′	As detailed for the sum of the reverse primers	n.a.
Cestode consensus real-time PCR 2				
Forward Primer	Ces1Gr2-4 F	5′-GGT-TTA-TTG-GAT-CRT-RCC-CGT-TAA-A-3′	*Dipylidium caninum*, *Diphyllobothrium latum*, *Diphyllobothrium ditrenum*, *Diphyllobothrium balaenopterae*, *Diphyllobothrium cameroni*, *Diphyllobothrium pacificum*, *Diplogonoporus grandis*, *Raillietina* spp., *Raillietina australis*, *Raillietina chiltoni*, *Raillietina sonini*	AB731643.1, MG582184.2, MG582181.1, MG582183.1, KF218246.1, KY552781.1, KF218250.1, KY552787.1, KY552792.1, KY552796.1, DQ925310.2, KF218253.1, KY945917.1, HG315734.1, AB353272.1, EU665464.1, EU665466.1, EU665467.1, AF286980.1, AY382313.1, EU665468.1
	Ces1Gr5 F	5′-TGG-TTT-ATT-GGA-TCR-TAC-TCG-TTA-AA-3′	*Hymenolepis diminuta*, *Hymenolepis nana*	AF124475.1, AF286983.1, JX310720.1, AF461124.1, AY193873.1, AY193874.1, AY193875.1
	Ces1Gr6-6a F	5′-GTT-TAT-TGG-ATC-GTA-CCC-GTT-AAR-3′	*Raillietina echinobothrida*, *Taenia saginata*, *Taenia solium*, *Taenia multiceps*, *Bertiella studeri*	MH119095.1, AB731616.1, JQ609338.1, DQ768166.1, U88076.1, AB731615.1, DQ157224.1, AB731621.1, GQ260089.1, GU323707.1
Reverse Primer	Ces1 A	5′-GGT-TGG-CTT-CTG-DTC-TAA-TAA-GTG-3′	As detailed for the sum of the forward primers	n.a.
Probe	Ces1 TM	5′-FAM-AGA-GCT-AAT-ACA-TGC-CHY-GAW-GCC-CTG-AC-BHQ1-3′	As detailed for the sum of the forward primers	n.a.
Combined positive-control plasmid sequence insert	Eco-R1-restriction-site-ATTGAAAACATTACGTAACTGGGAGTGAAAATTGCAATTATTTTTCATGAACGAGGAATTCCAAGTAAACGTAAGTCATTAGCTTACATTGATTACGTCCCTGCCCTTTGTACACACCGCCCGTCGCTGCCCGGAACTGAGCAATATCCAGAGGCAGGAAGAGATGTAATAA- Eco-R1-restriction-site-AATACGGATACGGGACTCACTAGAGGCTCCGTAATTCGAATGAGTACAATTTAAATCCTTTAACGAGGATCAACTGGAGGGCAAGTCTGGTGCCAGCAGCCGCGGTAACTCCAGCTCCAGAAGCGTATATTAAAGTTGTTGCAGTTCAAAAGCTCGTAGTTGGATCTGGGTCGCATGGCTACATGCCGTCGCTCGTGGGTCTGGCCTGGTTAC- Eco-R1-restriction-site-CTATGGTTTATTGGATCGTACCCGTTAAATGGGTAACTGTAATAACTCTAGAGCTAATACATGCCCCGATGCCCTGACTCTGTTAGCCTGCTGCTGCTTGCTTGTGTGTGGGTGGTGGCGGGTAGGCAGGGTGTGGGTGCACTTATTAGATCAGAAGCCAACCAACTGCTCGAACGTTGCGTGTGCAGTCCACTGCTGTAGGCGTGCGTGCGGGTGTTGAGAGGAGACCGCTTCTGGTGACTCTGGATAATTGTTACAGATCGCAGTCGGCCTTGAGTCGGCGACGGGTCCTTCAA- Eco-R1-restriction-site			

The real-time PCRs were run in 20 μL reaction volumes containing 10 μL HotStar master mix (Qiagen, Hilden, Germany), 5.0 mM total MgCl_2_, 2.5 × 10^−7^ mol of each primer, 6 × 10^−6^ mol probe, and 2.0 μL DNA eluate on a RotorGene 6000 cycler (Qiagen, Hilden, Germany). The steps were activation at 95 °C for 15 min followed by 45 cycles of 15 s denaturation at 95 °C, 30 s annealing at 60 °C and 30 s elongation at 72 °C. After this, the tubes were cooled to 40 °C for an additional 20 s before the run was finished. Note that n.a. = not applicable.

**Table 3:** Ct values of reactions and cross-reactions of group specific helminth real-time PCRs with nucleic acid extractions of helminths.

Sample Number	Species	Ct Values of Nematode Consensus Real-Time PCR 2	Ct Values of Trematode Consensus Real-Time PCR 2	Ct Values of Cestode Consensus Real-Time PCR 2	Ct Values of Nematode Consensus Real-Time PCR 1	Ct Value of Trematode Consensus Real-Time PCR 1	Ct Value of Cestode Consensus Real-Time PCR 1
TiHo1	*Trichinella spiralis*	17			42		
TiHo2	*Fasciola hepatica*		17		39 *	14	
TiHo3	*Toxocara canis*	13			14	24	
TiHo4	*Toxacara cati*	19	34 *		21	26 *	
TiHo5	*Taenia saginata*	26 *	34 *	32	37 *	30 *	26
TiHo7	*Angiostrongylus vasorum*	27	36 *		19		
TiHo9	*Dipylidium caninum*			23			13
Nr. 15	*Schistosoma mansoni*		12			11	
Nr. 16	*Strongyloides ratti*	32			14		

* Cross-reaction.

**Table 4:** Sensitivity and specificity of the group specific helminth real-time PCRs as calculated from the results of 96 residual DNA samples extracted from stool of patients with previous real-time PCR-based diagnosis of enteric helminth infections (see also Appendix A, Table A1).

Sensitivity/ Specificity	Nematode Consensus Real-Time PCR 2	Trematode Consensus Real-Time PCR 2	Cestode Consensus Real-Time PCR 2	Nematode Consensus Real-Time PCR 1	Trematode Consensus Real-Time PCR 1	Cestode Consensus Real-Time PCR 1
Sensitivity in % (n/n)	40% (20/50)	84.6% (11/13)	62.9% (22/35)	30% (15/50)	76.9% (10/13)	85.7% (30/35)
Specificity in % (n/n)	90.6% (87/96)	99.0% (95/96)	97.9% (94/96)	94.8% (91/96)	100% (96/96)	94.8% (91/96)

**Table 5:** Summary of results of helminth real-time PCRs with microscopically positive samples (*n* = 37) after extraction using the QiaAMP DNA Stool Mini Kit. Sensitivity and specificity were calculated in comparison with the microscopical results of the stool samples.

PCR Target	Sensitivity	Specificity	Mean Ct Value	Standard Deviation (SD) ^5^	Median Ct Value
Pan-trematode 18S rRNA gene	60% (3/5)	96.9% (31/32) ^1^	25.3	2.6	25.0
Pan-trematode 28S rRNA gene	60% (3/5)	96.9% (31/32) ^1^	26.8	1.3	27.0
Pan-cestode 18S rRNA gene	100% (3/3)	100% (34/34)	27.7	1.5	28.0
Pan-cestode cox gene	100% (3/3)	97.1% (33/34) ^2^	32.3	2.6	31.5
Pan-nematode 18S rRNA gene	20.7% (6/29)	100% (8/8)	32.5	5.3	35.0
Pan-nematode cox gene	41.4% (12/29)	100% (8/8)	32.6	3.4	33.5
*Ascaris lumbricoides*	40% (2/5)	100% (32/32)	32.5	0.7	32.5
*Ancylostoma* spp.	100% (2/2)	100% (32/32)	31.5	0.7	31.5
*Necator americanus*	100% (3/3)	100% (32/32)	27.3	4.5	27.0
*Strongyloides stercoralis*	38.5% (5/13)	100% (24/24)	29	4.4	28
*Schistosoma* spp.	75.0% (3/4)	96.7% (29/30) ^3^	18.5	2.6	18
*Trichurius trichiura*	33.3% (1/3)	100% (31/31)	27	n.a.	27
*Taenia saginata*	n.a. (0/0)	100% (34/34)	n.a.	n.a.	n.a.
*Taenia solium*	n.a. (0/0)	100% (34/34)	n.a.	n.a.	n.a.
*Enterobius vermicularis*	50% (1/2)	96.9% (31/32) ^4^	27	1.7	26
*Hymenolepis nana*	100% (3/3)	100% (32/32)	30	5.7	30

n.a. = not applicable. ^1^ Reaction with a sample with *Trichuris* eggs; ^2^ reaction with a sample with *Ascaris* and *Trichuris* eggs; ^3^ reaction with a sample with *Trichuris* eggs; ^4^ reaction with a sample with *Ascaris* eggs. Concordant cross-reaction of ^1^ and ^3^ makes the microscopical nondetection of *Schistosoma* eggs likely in this sample. ^5^ SD values with low absolute numbers of samples have to be interpreted with care.

**Table 6:** Nucleic acid extraction scheme comparison using the scheme without bead-beating and the scheme with bead-beating on 77 stool samples with the group specific helminth real-time PCRs.

Genus/Species	Extraction Method	Number *n*	Number of Positives (%)	*p*-Value ^1^	Ct-Value Mean (±Standard Deviation SD)	*p*-Value ^2^	Cohen’s Kappa (0.95 Confidence Interval CI)
18S rRNA gene-based pan-cestode real-time PCR	Without bead beating	77	20 (26)	0.476	31.1 (3.5)	0.361	0.683
	With bead beating	77	24 (31)		20.0 (4.2)		(0.503, 0.863)
cox gene-based pan-cestode real-time PCR	Without bead beating	77	25 (32)	0.864	35.3 (4.5)	0.093	0.853
	With bead beating	77	26 (34)		33.3 (4.2)		(0.729, 0.977)
18S rRNA gene-based pan-trematode real-time PCR	Without bead beating	77	9 (12)	1	28.2 (2.3)	0.588	0.874
	With bead beating	77	9 (12)		29.2 (4.9)		(0.703, 1)
28S rRNA gene-based pan-trematode real-time PCR	Without bead beating	77	9 (12)	0.797	30.4 (2.0)	0.071	0.802
	With bead beating	77	8 (10)		28.3 (2.4)		(0.585, 1)
18S rRNA gene-based pan-nematode real-time PCR	Without bead beating	77	29 (38)	0.621	32.1 (3.3)	0.223	0.756
	Without bead beating	77	32 (42)		31.1 (3.3)		(0.607, 0.905)
cox gene-based pan-nematode real-time PCR	Without bead beating	77	18 (23)	0.4623	32.4 (2.9)	0.775	0.702
	With bead beating	77	22 (29)		32.1 (4.5)		(0.524, 0.881)

^1^ Tests of proportions. ^2^ Unpaired T-test after nonsignificant testing (*p* value > 0.05) for the equality of variances.

**Table 7:** Nucleic acid extraction scheme comparison using the scheme without bead-beating and the scheme with bead-beating on 77 stool samples with the genus- and species-specific in-house helminth real-time PCRs.

Genus/Species	Extraction Method	Number *n*	Number of Positives (%)	*p*-Value ^1^	Ct-Value Mean (±Standard Deviation SD)	*p*-Value ^2^	Cohen’s Kappa (0.95 Confidence Interval CI)
house *Ancyclostoma* spp. real-time PCR	Without bead beating	77	1 (1)	1	30	n.e.	1 (1, 1)
	With bead beating	77	1 (1)		29		
in-house *Strongyloides stercoralis* real-time PCR	Without bead beating	77	11 (14)	0.113	34.2 (3.3)	0.076 *	0.176
	With bead beating	77	5 (6)		26.0 (7.8)		(−0.113, 0.466)
in-house *Necator americanus* real-time PCR	Without bead beating	77	7 (9)	0.006	34.4 (4.2)	0.070	0.272
	With bead beating	77	20 (26)		31.6 (3.2)		(0.040, 0.505)
in-house *Ascaris lumbricoides* real-time PCR	Without bead beating	77	3 (4)	0.043	30.3 (1.5)	0.842	−0.064
	With bead beating	77	10 (13)		30.6 (2.1)		(−0.136, 0.008)
in-house *Trichuris trichiura* real-time PCR	Without bead beating	77	14 (18)	0.547	29.6 (3.2)	0.330	0.476
	With bead beating	77	17 (22)		30.7 (2.8)		(0.233, 0.719)
in-house *Taenia solium* real-time PCR	Without bead beating	77	2 (3)	0.405	27.0 (1.4)	0.043	0.655
	With bead beating	77	4 (5)		31.0 (1.6)		(0.211, 1)
in-house *Schistosoma* spp. real-time PCR	Without bead beating	77	10 (13)	0.616	23.3 (4.5)	0.470	0.874
	With bead beating	77	8 (10)		21.8 (4.3)		(0.704, 1)
in-house *Taenia saginata* real-time PCR	Without bead beating	77	1 (1)	n.e.	18 (n.e.)	n.e.	n.e.
	With bead beating	77	0		n.a.		
in-house *Hymenolepis nana* real-time PCR	Without bead beating	77	25 (32)	0.864	28.9 (3.7)	0.335	0.912
	With bead beating	77	26 (34)		27.8 (4.4)		(0.815, 1)
in-house *Enterobius vermicularis* real-time PCR	Without bead beating	77	2 (3)	0.146	31.5 (0.7)	0.6242	0.220
	With bead beating	77	6 (8)		32.7 (3.0)		(−0.176, 0.615)
in-house *Schistosoma mansoni* real-time PCR	Without bead beating	77	10 (13)	0.374	24.4 (4.7)	0.094 *	0.705
	With bead beating	77	14 (18)		29.8 (10.0)		(0.486, 0.924)
in-house *Schistosoma haematobium* real-time PCR	Without bead beating	77	0	n.e.	n.a.	n.e.	n.e.
	With bead beating	77	0		n.a.		

^1^ Tests of proportions. ^2^ Unpaired T-test after nonsignificant testing (*p* value > 0.05) for the equality of variances. * Unpaired T-test with unequal variances after significant testing for the equality of variances (*p* value ≤ 0.05). n.a. = not applicable. n.e. = nonestimable.

**Table 8:** Nucleic acid extraction scheme comparison using the scheme without bead-beating and the scheme with bead-beating on 77 stool samples with the genus- and species-specific commercial Tib MolBiol helminth real-time PCRs.

Genus/Species	Extraction Method	Number *n*	Number of Positives (%)	*p*-Value ^1^	Ct-Value Mean (±Standard Deviation SD)	*p*-Value ^2^	Cohen’s Kappa (0.95 Confidence Interval CI)
Tib MolBiol *Ascaris lumbricoides* real-time PCR	Without bead beating	51	4 (8)	0.010	34.0 (2.8)	0.015	0.558
	With bead beating	77	14 (18)		30.2 (2.4)		(0.176, 0.941)
Tib MolBiol *Strongyloides stercoralis* real-time PCR	Without bead beating	51	3 (6)	0.508	30.7 (2.5)	0.746	0.847
	With bead beating	77	7 (9)		29.3 (6.7)		(0.553, 1)
Tib MolBiol *Necator americanus* real-time PCR	Without bead beating	51	9 (18)	0.045	34.2 (3.0)	0.001	0.582
	With bead beating	77	26 (34)		29.5 (3.5)		(0.308, 0.857)
Tib MolBiol *Ancylostoma* spp. real-time PCR	Without bead beating	51	1(2)	0.7675	33.00	n.e.	1
	With bead beating	77	1 (1)		32.00		(1, 1)
Tib MolBiol *Hymenolepis nana* real-time PCR	Without bead beating	45	7 (16)	0.041	32.3 (2.4)	0.003	0.588
	With bead beating	77	25 (32)		26.6 (4.4)		(0.300, 0.877)
Tib MolBiol *Enterobius vermicularis* real-time PCR	Without bead beating	45	2 (4)	0.063	32.5 (0.7)	0.8012	0.237
	With bead beating	77	12 (16)		31.8 (3.5)		(−0.206, 0.680)
Tib MolBiol *Schistosoma* spp. real-time PCR	Without bead beating	45	9 (20)	0.085	29.3 (2.5)	<0.001	0.848
	With bead beating	77	7 (9)		22.1 (1.5)		(0.646, 1)

^1^ Tests of proportions. ^2^ Unpaired T-test after nonsignificant testing (*p* value > 0.05) for the equality of variances. n.e. = nonestimable.

**Table 9:** Nucleic acid extraction scheme comparison using the scheme without bead-beating and the scheme with bead-beating with real-time PCRs for enteropathogenic bacteria and enteric protozoa on 67 stool samples.

Genus/Species	Extraction Method ^#^	Number *n*	Number of Positives (%)	*p*-Value ^1^	Ct-Value Mean (±Standard Deviation SD)	*p*-Value ^2^	Cohen’s Kappa (0.95 Confidence Interval CI)	
In-house *Salmonella* spp. real-time PCR	Without bead beating	67	3 (4)	0.649	31.3 (2.1)	0.334	0.793	
	With bead beating	67	2 (3)		29.5 (0.7)		(0.398, 1)	
In-house *Yersinia* spp. real-time PCR	Without bead beating	67	0	n.e.	n.a.	n.e.	n.e.	
	With bead beating	67	0		n.a.			
In-house *Campylobacter jejuni* real-time PCR	Without bead beating	67	12 (18)	0.819	25.8 (3.2)	0.578	0.633	
	With bead beating	67	11 (16)		26.5 (3.6)		(0.384, 0.882)	
In-house *Shigella* spp./enteroinvasive *Escherichia coli* real-time PCR	Without bead beating	67	2 (3)	<0.001	31.0 (1.4)	0.821	0.179	
	With bead beating	67	16 (24)		31.6 (3.7)			
							(−0.040, 0.397)	
In-house *E. histolytica* real-time PCR	Without bead beating	67	0	n.e.	n.a.	n.e.	n.e.	
	With bead beating	67	1 (1)		33 (n.e.)			
In-house *Cyclospora* spp. real-time PCR	Without bead beating	67	6 (9)	0.572	30.8 (2.9)	0.059 *	0.045	
	With bead beating	67	8 (12)		33.8 (0.9)		(−0.223, 0.313)	
In-house *Giardia duodenalis* real-time PCR	Without bead beating	67	19 (28)	1	28.7 (3.9)	0.194	0.706	
	With bead beating	67	19 (28)		27.1 (3.7)		(0.517, 0.895)	
In-house *Cryptosporidium* spp. real-time PCR	Without bead beating	67	0	n.e.	n.a.	n.e.	n.e.	
	With bead beating	67	1 (1)		33.0 (n.e.)			

^1^ Tests of proportions. ^2^ Unpaired T-test after nonsignificant testing (*p* value > 0.05) for the equality of variances. * Unpaired T-test with unequal variances after significant testing for the equality of variances (*p* value ≤ 0.05). n.a. = not applicable. n.e. = nonestimable.

## Data Availability

All relevant data are provided within the manuscripts and its tables. Raw data can be made available on reasonable request.

## Appendix A

**Table A1:** Details of the assessment of group specific helminth real-time PCRs with residual DNAs of stool samples tested positive with genus- or specific helminth real-time PCR (see also Table 4).

Sample Number	Species/Genus According to in-House Real-Time PCR (Ct Value)	Expected Consensus Real-Time PCR Result	Ct Values of Nematode Consensus Real-Time PCR 2	Ct Values of Trematode Consensus Real-Time PCR 2	Ct Values of Cestode Consensus Real-Time PCR 2	Ct Values of Nematode Consensus Real-Time PCR 1	Ct Value of Trematode Consensus Real-Time PCR 1	Ct Value of Cestode Consensus Real-Time PCR 1
1	*Hymenolepis nana* (25)	Cestode			29			33
2	*Hymenolepis nana* (27)	Cestode						36
3	*Hymenolepis nana* (29)	Cestode			32			34
4	*Hymenolepis nana* (27)	Cestode	30		30			35
5	*Hymenolepis nana* (32)	Cestode			33			33
6	*Hymenolepis nana* (23)	Cestode			27			29
7	*Hymenolepis nana* (33)	Cestode			36			39
8	*Trichuris trichiura* (30)	Nematode	32					
9	*Hymenolepis nana* (29)	Cestode			31			34
10	*Trichuris trichiura* (33)	Nematode	36					
11	*Hymenolepis nana* (29)	Cestode			32			36
12	*Trichuris trichiura* (24)	Nematode	30					
13	*Hymenolepis nana* (29)	Cestode						33
14	*Hymenolepis nana* (29)	Cestode			33			34
15	*Schistosoma* spp. (34)	Trematode						
16	*Ascaris lumbricoides* (29)	Nematode						
17	*Trichuris trichiura* (32)	Nematode	32			32		
18	*Trichuris trichiura* (26)	Nematode	30					
19	*Taenia solium* (26)	Cestode			30			29
20	*Necator americanus* (36)	Nematode	35					
21	*Trichuris trichiura* (27)	Nematode				34		
22	*Hymenolepis nana* (29), *Enterobius vermicularis* (36)	Nematode, cestode	29		32			32
23	*Necator americanus* (34)	Nematode	29			31		33
24	*Ascaris lumbricoides* (30)	Nematode	31			35		
25	*Hymenolepis nana* (30)	Cestode	33					39
26	*Trichuris trichiura* (29)	Nematode	32					
27	*Necator americanus* (36)	Nematode	30			29		
28	*Ascaris lumbricoides* (32)	Nematode	41			32		
29	*Hymenolepis nana* (25)	Cestode	37		30			33
30	*Hymenolepis nana* (32)	Cestode	31			30		40
31	*Hymenolepis nana* (31)	Cestode						38
32	*Hymenolepis nana* (29)	Cestode	30		35			38
33	*Enterobius vermicularis* (32)	Nematode	32			29		
34	*Taenia solium* (28)	Cestode	28			34		
35	*Strongyloides stercoralis* (31), *Necator americanus* (37)	Nematode	32			42		
36	*Trichuris trichiura* (27)	Nematode	32			32		
37	*Trichuris trichiura* (25)	Nematode	32			32		
38	*Ascaris lumbricoides* (28)	Nematode						
39	*Hymenolepis nana* (29)	Cestode	31					
40	*Trichuris trichiura* (28)	Nematode	32			31		
41	*Trichuris trichiura* (27)	Nematode	32			30		
42	*Trichuris trichiura* (28)	Nematode	31			35		35
43	*Hymenolepis nana* (25)	Cestode	31			35		35
44	*Hymenolepis nana* (30)	Cestode	28		28	34		
45	*Trichuris trichiura* (31)	Nematode						
46	*Hymenolepis nana* (17)	Cestode			20			29
47	*Taenia saginata* (18)	Cestode						
48	*Enterobius vermicularis* (33)	Nematode						
49	*Strongyloides stercoralis* (37)	Nematode						
50	*Necator americanus* (26)	Nematode	29					
51	*Necator americanus* (38)	Nematode						
52	*Strongyloides stercoralis* (33)	Nematode						
53	*Strongyloides stercoralis* (41)	Nematode						
54	*Strongyloides stercoralis* (35)	Nematode				39		
55	*Hymenolepis nana* (30)	Cestode				36		
56	*Strongyloides stercoralis* (36)	Nematode						
57	*Enterobius vermicularis* (31)	Nematode						
58	*Strongyloides stercoralis* (34)	Nematode						
59	*Strongyloides stercoralis* (36)	Nematode						
60	*Strongyloides stercoralis* (37)	Nematode						
61	Strongyloides stercoralis (36)	Nematode						
62	*Schistosoma* spp. (20), *Strongyloides stercoralis* (40)	Trematode, nematode		26			27	
63	*Schistosoma* spp. (26)	Trematode		33			35	
64	*Schistosoma* spp. (22)	Trematode		30			30	
65	*Hymenolepis nana* (30)	Cestode			32			40
66	*Schistosoma* spp. (17)	Trematode		24			28	
67	*Schistosoma* spp. (28)	Trematode		35				
68	*Schistosoma* spp. (23)	Trematode		28			31	
69	*Hymenolepis nana* (27)	Cestode			30			36
70	*Strongyloides stercoralis* (36)	Nematode						
71	*Hymenolepis nana* (30)	Cestode						
72	*Hymenolepis nana* (29)	Cestode			32			37
73	*Schistosoma* spp. (23)	Trematode		29			34	
74	*Schistosoma* spp. (20)	Trematode		26			29	
75	*Schistosoma* spp. (22)	Trematode		27	36		29	44
76	*Hymenolepis nana* (31)	Cestode						43
77	*Hymenolepis nana* (29)	Cestode			32			38
78	*Hymenolepis nana* (32)	Cestode			34			43
79	*Hymenolepis nana* (35)	Cestode			36			37
80	*Schistosoma* spp. (26)	Trematode		31			33	
81	*Hymenolepis nana* (32)	Cestode			32			35
82	*Strongyloides stercoralis* (35)	Nematode						
83	*Hymenolepis nana* (31)	Cestode			34			
84	*Schistosoma* spp. (21)	Trematode			28			29
85	*Strongyloides stercoralis* (34)	Nematode						
86	*Trichuris trichiura* (34)	Nematode						
87	*Strongyloides stercoralis* (33)	Nematode						47
88	*Strongyloides stercoralis* (31)	Nematode			37			
89	*Strongyloides stercoralis* (32)	Nematode						
90	*Necator americanus* (25)	Nematode		26		30		
91	*Strongyloides stercoralis* (31)	Nematode						
92	*Enterobius vermicularis* (36)	Nematode						
93	*Schistosoma* spp. (32)	Trematode						
94	*Strongyloides stercoralis* (32)	Nematode						
95	*Ascaris lumbricoides* (39)	Nematode						
96	*Strongyloides stercoralis* (32)	Nematode						45

Potentially falsely positive results as compared with genus- or species-specific real-time PCR are highlighted in gray, hypothetically falsely negative results as compared with genus- or species-specific real-time PCR are indicated by a black field.
